# Domestic Correspondence

**Published:** 1890-05

**Authors:** W. C. Barrett


### Domestic Correspondence.

---

To the Editor:

*A question of professional ethics.*—I have never been of those who felt particularly solicitous concerning the source of dental literature, so long as the quality was satisfactory. Particularly in our journalism have I felt indifferent, provided it be good journalism, and such as will add to the dignity and subserve the best interests of dentistry. Perhaps I have at times thought that I recognized a tendency in the mercantile and trade element to dictate to and antagonize the professional, but I have clung to the idea that these matters will regulate themselves. I have thought that only fair play was demanded, and the good sense of dentistry would settle the rest.

Our calling is not strictly amenable to the rules which govern any other, because, while, as I believe, it is professional in its main aspect, it has a mechanical and artisan-like side. Every thinking man knows that the laws which govern trade and mechanics are not applicable to a profession. The methods which are entirely legitimate in mercantile ethics are inadmissible where a liberal profession is concerned. Advertising, for instance, which is the life of trade, all men agree is quite beyond the bounds of professional ethics.

The dividing line between that which is "professional" and that which is "unprofessional" is exceedingly difficult to make manifest to the eye of a layman, but it is as obvious as one's nose to him who, having been regularly admitted within the pale, is animated by true professional feeling. To men engaged in trade, and whose minds are pervaded by mercantile ideas, the laws of professional ethics are quite incomprehensible. An American or a Frenchman cannot understand the admiration of the Welchman for the odorous leek; but to him who has been trained in Welch lore and tradition the leek stands for all that is honorable and heroic in his national history. To the Indian our national flag is but a piece of gaudy patchwork; but to the American patriot it is the symbol of his nationality. So to the real professional man the spirit of the code of ethics, although it may be foolishness to the unprofessional Greek, is that which marks his professionality.

I recognize in dentistry two elements; the one strictly professional according to the code of our mother profession, medicine; the other more nearly allied to mechanics and trade. Although in a measure antagonistic in certain aspects, it does not follow that they should make war upon each other. Their mutual relations may, and should be, discussed in a spirit of temperance and candor. Good men on each side are doubtless swayed by their interests and their associations, but that gives no excuse for recrimination. The man who has, perhaps, gone through a professional course of study and then left the professional ranks and gone into trade, or who has introduced some manufacturing interest into his professional life, cannot but be imbued with the spirit of trade. Another may have devoted himself to the mechanical part of his calling and drifted out of professional associations and sympathies. He may have an inventive talent, and have devised some ingenious appliance from which he desires to reap a benefit. There is no reason why he should not do this, so long as it is done decently and in order, but he should not insist that the man who has kept strictly within ethical lines should adopt his views and methods. He should be prompt to recognize the fact that just to the extent to which he has gone into trade has he abandoned a professional life, for a man cannot follow both. Who will claim that the dentist who has commenced the manufacture and sale of some vendible article, or who has gone into mechanics, is any better judge of ethical matters than he who has kept within the line of medical ethics? More than this, who will deny that the mind of such a man is not, to a greater or less degree, imbued with those trade methods and ideas which are antagonistic to professional life? Such a man may be quite as conscientious as another, but he is scarcely qualified to sit in judgment upon professional matters. Trade is quite as honorable as a profession, but it is not identical with it, and is not amenable to the same laws. If a man is imbued with the spirit of the one, he cannot enter into the feelings of the other.

The most of our dental journals are edited by men engaged in active professional life. Their sympathies are with dentistry and not with that which is antagonistic to it. They are legitimate members of the profession, and not outside of it, engaged in trade. So when a question of ethics arises, they are qualified to speak upon it without being liable to the charge of impertinent intermeddling with that with which they have nothing to do, and which, from the very circumstances of the case, they cannot comprehend. Yet some, even of these, it seems to me, have not been professionally just in certain instances.

Nearly a year ago, Dr. H. C. Merriam presented before a well-known dental society his views upon certain matters of professional ethics. No one could question his right to do this, for he occupies at least a respectable position among dentists, is known as a candid and honorable man, while his intelligence is undisputed. Certainly no one will deny his devotion to what he believes to be the best interests of dentistry, nor the purity of his motives. He viewed matters, as might naturally be expected, from an exalted professional stand-point. He thought he saw certain tendencies which were inimical to professional advancement, and he pointed these out, clearly but dispassionately. There was no tinge of personal malice, no bitterness of disappointed ambition manifest, but from his high position he argued against what he thought were debasing tendencies. What was the consequence? The journal in which his article was published as a part of the transactions of the society made answer to it from the mercantile view, as of course was to be expected, but it also took him personally to task for openly discussing general principles. The very party which is least qualified to speak upon a question of ethics severely condemned the writer for discussing professional relations. An advocate who has no standing whatever in court assumed to sit as a judge in a strictly professional matter, and this was echoed not only by every editor who is in trade, but by some from whom might have been expected different sentiments. "Tray, Blanche, and Sweetheart" all opened upon the trail of a professional man who had only spoken of professional matters before a professional audience. There seemed a determination to make an example of him; to so hold him up to personal ridicule and disparagement as to make of him a solemn warning. A distinct opposition to the commonly accepted ethical spirit was shadowed forth, and a determination to deter others from resisting what they might honestly believe to be an encroachment of the mercantile upon the professional spirit. Granted that the writer was utterly mistaken in his views of professional matters; had he no right to express his opinions before his professional brethren and invite discussion of the subject? Perhaps he was incompetent to judge what was for the best interests of dentistry; but were those who had nothing in common with professional life any better qualified? If he misrepresented the real feelings of true professional men, did he not appear before a tribunal which is an acknowledged authority upon such questions?

The treatment which he received stirred the blood of more than one dentist who had not recognized a possible danger, and instead of repressing a professional spirit it has stimulated it as nothing else could have done. It seems time that some voice should be raised in behalf of fair play. If professional men are to be throttled because they express views which may not tend to the profit of trade, we all should know it. We have seen dentistry bound hand and foot, by patent rights and sordid monopolistic aggression, in a vulcanite web from which nothing but death relieved it. We are even now threatened by another exhibition of this same tendency, and nothing but a bold, determined, united action will protect us. That same patent-right element has endeavored to strike down the man who stands as our representative in resistance. In his defence every professional arm has been raised. Why should not the same desire for fair play be guaranteed another who honestly endeavors to strike a blow for what he deems to be professional honor? If he be mistaken, we ourselves should be the judges of the fact. If he be right, surely it is time that somebody sounded the trumpet and raised the cry, "To your tents, O Israel!" We have no quarrel with trade, but we do protest against its assuming to sit as a judge in matters of professional ethics.

W. C. BARRETT.

